# Retrospective Analysis of Route Selection for Hysterectomy for Benign Indications at Ochsner Baptist Hospital

**DOI:** 10.31486/toj.20.0017

**Published:** 2020

**Authors:** Emily G. Blosser, George B. Morris, Rajiv B. Gala

**Affiliations:** ^1^ Department of Obstetrics and Gynecology, Ochsner Clinic Foundation, New Orleans, LA; ^2^ The University of Queensland Faculty of Medicine, Ochsner Clinical School, New Orleans, LA

**Keywords:** *Hysterectomy*, *hysterectomy–vaginal*, *minimally invasive surgical procedures*, *robotic surgical procedures*

## Abstract

**Background:** Hysterectomy, the most common gynecologic procedure in the United States, can be performed in a number of ways. A shift in surgical practice toward cost-effective and minimally invasive approaches provides an impetus to maximize early training in vaginal surgery for resident physicians.

**Methods:** A total of 62 abdominal, 303 robotic, and 41 vaginal hysterectomies performed between January 1, 2015 and December 31, 2017 at Ochsner Baptist Hospital in New Orleans, LA, that met inclusion criteria were retrospectively reviewed with a previously published route selection algorithm. We applied the algorithm using preoperative and postoperative data collected via medical record review to determine if our practices favor minimally invasive approaches.

**Results:** Analysis using preoperative variables identified 152 robotic cases that were vaginal hysterectomy candidates (50.2%). Postoperative analysis of the same cases identified 127 (41.9%) vaginal hysterectomy candidates. Among abdominal cases, 37 (59.7%) called for a less invasive approach by preoperative findings: 7 (11.3%) vaginal and 30 (48.4%) laparoscopic. The algorithm sorted only 25 of the 62 abdominal cases (40.3%) to the abdominal approach.

**Conclusion:** Use of a hysterectomy route selection algorithm preoperatively improves identification of candidates for minimally invasive hysterectomy.

## INTRODUCTION

Hysterectomy, the definitive treatment for many benign gynecologic pathologies, is one of the most common major gynecologic procedures performed in the United States. The different surgical approaches to hysterectomy have unique benefits and risks. Nationally, more than 50% of hysterectomies are performed through a large abdominal incision.^1^ While the American College of Obstetricians and Gynecologists (ACOG) recommends choosing the least invasive route, the transvaginal approach, a number of patient- and surgeon-specific factors influence route selection.^2-6^

Alternative minimally invasive approaches, such as standard and robotic laparoscopy, have gained attention during the last 2 decades. In 2000, the da Vinci Surgical System (Intuitive Surgical, Inc) became the first robotic surgical platform commercially available in the United States. In 2005, the US Food and Drug Administration approved the da Vinci Surgical System for use in gynecologic surgery.^7^ The robotic system was marketed as a clear improvement to standard laparoscopy in terms of ergonomics, 3-dimensional visualization, and hand- and wrist-like dexterity, and many hospitals nationwide quickly adopted it.^2,6^ Despite its perceived superiority to standard laparoscopy, the da Vinci Surgical System remains cost-prohibitive for many rural and community hospitals, with platform startup carrying a $1 to $2 million price tag.^3,4,8-11^ Hospital systems that have initiated robotics programs have prioritized robotic surgery to maintain their surgeons’ expertise and to improve the overall cost-effectiveness of the device.^3,10,12^ While robotic technology has reduced the number of open hysterectomies by approximately 20%, vaginal hysterectomies have also shown a downward trend overall.^13^

The downward trend in vaginal hysterectomies highlights not only the need for a paradigm shift in surgical planning but also the need for substantial and early training in minimally invasive surgical approaches, such as vaginal surgery.^14-17^ In 2018, the Accreditation Council for Graduate Medical Education (ACGME) revised its minimum numbers for resident education in obstetrics and gynecology to emphasize training in minimally invasive hysterectomy approaches vs laparotomy.^18^ Critical to the success of reprioritizing vaginal hysterectomy is highlighting its feasibility and cost-effectiveness relative to other approaches by implementing evidence-based algorithms that objectively identify candidates for vaginal surgery.^19-21^ Our hypothesis was that we could identify potentially missed vaginal hysterectomy candidates at Ochsner Baptist Hospital in New Orleans, LA, by using a nationally accepted algorithm.

## METHODS

The study was approved by the Ochsner Institutional Review Board. All authors were on staff at the institution at the time of data collection. The study was designed and executed as a retrospective medical record review of patients who underwent hysterectomy via abdominal, robotic, or vaginal routes for benign indications at Ochsner Baptist Hospital between January 1, 2015 and December 31, 2017. Sixteen general gynecology faculty surgeons were included in the study. Gynecologic oncologists and urogynecologists were excluded.

Laparoscopic-assisted vaginal hysterectomies, supracervical hysterectomies, cesarean hysterectomies, and minimally invasive hysterectomies that had to be converted intraoperatively were excluded. Additional exclusions are listed in Table 1.

**Table 1.** Exclusion Criteria

Adnexal disease as primary indication for surgery	Minimally invasive hysterectomy converted intraoperatively
Adnexal torsion	Müllerian or uterine anomalies
Age <18 years	Ovarian, fallopian tube, or primary peritoneal cancer
Cervical cancer more advanced than stage 1A1	Pelvic kidney
Cesarean hysterectomy	Planned appendectomy, cholecystectomy, or bowel surgery
Concomitant anti-incontinence procedures	Planned umbilical hernia repair affecting route choice
Concomitant pelvic organ prolapse operation other than pure uterine	Radical hysterectomy
Emergent hysterectomy	Risk-reducing surgery (ie, BRCA positive)
Endometrial hyperplasia of increased complexity	Supracervical hysterectomy
History of multiple cone excisions with no available cervical tissue	Tubo-ovarian abscess
Laparoscopic hysterectomy or laparoscopy-assisted vaginal hysterectomy	Uterine cancer or suspicion for sarcoma
Mesh-related surgery or excision	

Patient demographics, history, physical examination, laboratory and imaging results, operative reports, and postoperative care were manually extracted from electronic health records and entered into an Excel (Microsoft Corp) spreadsheet designed to summarize the perioperative period. Age, body mass index (BMI), surgical indication, parity, significant medical history, history of >1 laparotomy, imaging, and physical examination findings were summarized for evaluation of route selection based on preoperative findings. Pathology findings, documented adhesions, length of hospital stay, and postoperative complications and visits were catalogued for evaluation of route selection based on intraoperative and postoperative findings.

The algorithm detailed by Schmitt et al^15^ was used to retrospectively reclassify the optimal route of hysterectomy based on objective characteristics—preoperative pathology, vaginal accessibility, uterine size, and uterine weight—instead of surgeon preference. We defined vaginal accessibility by a history of ≥1 vaginal delivery and history of ≤1 laparotomy combined with physical examination findings of a uterus ≤12 weeks’ gestation size and adequate mobility. Deviations from the expected route were defined as in Schmitt et al.^15^

## RESULTS

During the 3-year study period, 1,077 hysterectomies were performed at Ochsner Baptist Hospital. A total of 194 abdominal cases were reviewed. Of those, 97 were performed by an alternative route or converted, 19 were supracervical hysterectomies, 14 were cesarean hysterectomies, and 2 were radical hysterectomies, leaving 62 abdominal cases available for analysis.

A total of 842 robotic hysterectomies were identified. Of those, 539 cases were excluded for the following reasons: 431 involved gynecologic oncology, 45 were performed by urogynecology, 38 had pelvic masses, 20 were combined procedures, 3 were converted to abdominal or laparoscopic approach, and 2 involved suspected injury related to the Essure device. The remaining 303 robotic cases were included and analyzed.

During the study period, 41 vaginal hysterectomies were performed that met inclusion criteria.

Demographics and baseline characteristics of the patients included in the analysis are summarized in Table 2.

**Table 2.** Demographic Characteristics of Patients by Surgical Approach

	Route of Hysterectomy		
Variable	Abdominal, n=62	Robotic, n=303	Vaginal, n=41
Age, years, mean ± SD	44.35 ± 5.88	44.7 ± 7.75	47 ± 10.36
Body mass index, kg/m^2^, mean ± SD	34.61 ± 7.34	32.46 ± 8.5	29.86 ± 6.74
Race/ethnicity			
Black/African American	56 (90.3)	164 (54.1)	16 (39.0)
Caucasian	6 (9.7)	130 (42.9)	22 (53.7)
Hispanic, nonwhite	–	5 (1.7)	2 (4.9)
Asian	–	4 (1.3)	1 (2.4)

Note: Data are presented as n (%) unless otherwise indicated.

After analysis with preoperative variables including patient BMI, medical history, parity, and history of laparotomy, along with physical examination findings and imaging, the algorithm identified 152 robotic cases that could have been vaginal hysterectomy candidates (50.2%). When the 303 robotic cases were analyzed with postoperative variables such as intraoperative adhesions and pathology weight and size, 127 (41.9%) were vaginal hysterectomy candidates.

Among the abdominal cases, the algorithm called for a less invasive approach based on preoperative findings in 37 (59.7%) cases: 7 (11.3%) vaginal and 30 (48.4%) laparoscopic based on preoperative findings. Additionally, almost 50% of cases performed abdominally—30 cases by preoperative analysis and 27 cases by postoperative analysis—were better laparoscopic candidates. The algorithm sorted only 25 of the 62 abdominal cases (40.3%) to the abdominal approach (Table 3).

**Table 3.** Booked Surgical Approach vs Route Selection With the Algorithm

	Optimal Route Selection by Preoperative Analysis			Optimal Route Selection by Postoperative Analysis		
Booked Surgical Approach	Abdominal	Laparoscopic	Vaginal	Abdominal	Laparoscopic	Vaginal
Abdominal, n=62	25 (40.3)	30 (48.4)	7 (11.3)	32 (51.6)	27 (43.5)	3 (4.8)
Robotic, n=303	19 (6.3)	132 (43.6)	152 (50.2)	3 (1.0)	173 (57.1)	127 (41.9)
Vaginal, n=41	0 (0)	7 (17.1)	34 (82.9)	0 (0)	2 (4.9)	39 (95.1)

Note: Data are presented as n (%).

We sought to verify the algorithm recommendations using postoperative findings. The algorithm overestimated the number of potential vaginal hysterectomy candidates who were better served with a planned robotic approach (152 vs 127 cases, accounting for 25 misclassifications, 8.3%), primarily because of previously undiagnosed pelvic adhesive disease, endometriosis, or inaccurate estimates of uterine size with physical examination or imaging. In addition, 3 robotic cases and 4 vaginal cases would have been appropriated to the abdominal approach by intraoperative and postoperative findings (7 misclassified cases, 11.3%).

Conversely, the algorithm at times called for more invasive surgery than was necessary. Sixteen of the 19 robotic cases recommended for an abdominal approach were correctly performed robotically after reviewing postoperative findings.

## DISCUSSION

While the ACOG recognizes 3 acceptable routes of hysterectomy, the College makes no specific or standard recommendations for route selection but instead recommends tailoring route selection to each patient in a way that favors a minimally invasive approach. At Ochsner Baptist Hospital, less invasive routes than the abdominal approach are prioritized. The algorithm shows the greatest utility in the objective selection of a minimally invasive route. Little evidence in the current literature suggests that any minimally invasive approach far outperforms the others in terms of patient recovery, tolerance, and satisfaction. The primary difference between these approaches is thought to be cost. An analysis of cost across all routes of hysterectomy at Ochsner hospitals in Louisiana is ongoing, but we anticipate that implementation of this algorithm and improved route selection will have a significant impact on cost, both to patient and hospital.

Our study demonstrates continued opportunities for optimization of route selection when applied to planned abdominal and robotic hysterectomies. The abdominal cases that could be sorted to a less invasive approach were more likely to sort to a laparoscopic approach than to a vaginal approach (Table 3). However, implementing this algorithm has identified some potential vaginal cases that were missed in favor of an overly invasive abdominal approach. While so few cases are unlikely to drastically alter resident training, they may have a significant impact on those patients’ experience with hysterectomy.

As expected, the algorithm identified many new potential vaginal cases. If all of the abdominal and robotic cases that the algorithm identified as potential vaginal cases had been attempted vaginally, the total number of vaginal hysterectomies from January 1, 2015 to December 31, 2017 would have increased from 41 to 171 based on postoperative analysis, with only 2 needing to be converted to a laparoscopic/robotic case (1.2% of 171). This change would most certainly have a positive impact on resident training in vaginal surgery and would also improve residents’ ability to achieve their ACGME minimally invasive hysterectomy numbers more expeditiously. Although it was not done for this study, another important analysis will be to examine standard laparoscopy cases for the same 3-year period. We suspect that the algorithm will retrospectively sort a number of additional cases from the laparoscopy pool to the vaginal approach. This review is underway. A prospective approach with standardization of preoperative physical examinations and documentation would also be beneficial in optimizing the algorithm for our hospital system.

One limitation to this study is that the algorithm may overstate the appropriateness of the vaginal approach, primarily because unforeseen intraoperative complications (such as pelvic adhesions, endometriosis, or anatomic abnormalities) may be undiagnosed until the day of surgery. However, the importance of our data showing that many abdominal cases would have sorted to a less invasive approach using the algorithm should be emphasized, as the data suggest that implementing the algorithm might improve patient access to less invasive routes of hysterectomy.

Retrospective medical record review is another limitation. Documentation of preoperative physical examinations was inconsistent. Consequently, potential vaginal cases were possibly overestimated or underestimated, as best judgment was used in the absence of a clear preoperative physical examination. Additionally, morcellation of the uterus made postoperative pathology challenging to assess for size. In such instances, the weight of the specimen was used to evaluate and select an approach, adding subjectivity to the study approach.

Despite these limitations, the study provides compelling evidence that implementation of an algorithm for sorting surgical approaches improves route selection and identifies a greater number of potential vaginal cases.

## CONCLUSION

Use of a hysterectomy route selection algorithm preoperatively can improve identification of candidates for minimally invasive hysterectomy and enhance resident training in vaginal surgery.

## Notes

This article meets the Accreditation Council for Graduate Medical Education and the American Board of Medical Specialties Maintenance of Certification competencies for Patient Care, Medical Knowledge, and Practice-Based Learning and Improvement.
